# On the Value of Tar in Bronchial Catarrh and Winter-Cough

**Published:** 1875-12

**Authors:** Sidney Ringer, Wm. Murrill

**Affiliations:** Professor of Materia Medica and Therapeutics in University College, London


### ON THE VALUE OF TAR IN BRONCHIAL CATARRH AND WINTER-COUGH.

By Sidney Ringer, M. D., Professor of Materia Medica and Therapeutics in University College, London; and Wm. Murrill, L. R. C. P.

The frequent and popular use of this remedy, both by the profession and the laity, in France and Belgium, led us to try its effects. Patients so susceptible to cold that they were obliged to remain indoors the whole winter, informed us that this remedy curtailed considerably the duration and lessened the severity of their catarrhal attacks, and that, by an occassional recourse to the tar, they became less prone to catch cold, and could more freely expose themselves to the weather without incurring an attack. It will be seen that our observations confirm these statements.

We employed tar in two grain doses, made into a pill, every three or four hours. From October to January, inclusive, we carefully watched its effects on twenty-five patients, whose ages varied from 34 to 70, the average being 44. All these patients had suffered for several years from winter-cough, lasting the whole winter. They were out-patients, and visited the hospital weekly, or oftener. Most of them were much exposed to the weather, while some were so ill that they were obliged to stop work, and, therefore, less exposed.

These patients suffered from the symptoms common in winter-cough—paroxysmal and violent cough, the paroxysms lasting from two to ten minutes, and recurring ten to twelve times a day, and, in the night, breaking their rest. The expectoration, frothy and slightly purulent, was generally rather abundant, amounting in some cases to half a pint or more in the day. The breathing was very short on exertion, but most could lie down at night without propping. The physical signs showed a variable amount of emphysema, with sonorous and sibilant rhonchus, and occasionally a little bubbling rhonchus at the base.

These patients usually began to improve from the fourth to the seventh day; the improvement rapidly increased, and, in about three weeks, they were well enough to be discharged. The improvement was so decided, that the patients returned to their work; even those who in previous years had been confined to the house the whole winter. The cough and expectoration improved before the breathing. In several cases the expectoration increased during the first three or four days; but its expulsion became easier, and with the improvement in the cough and expectoration, appetite and strength returned.

On discontinuing the tar, a relapse often occured in a week or two, and the patients returned with a request for more of the same medicine, and then, a second time, the symptoms quickly subsided. We found it useless in bronchial asthma, and its effects were more evident in cases where expectoration and cough were more marked than dyspnœa.

We have no doubt that tar is a good, useful, though, perhaps, not a striking remedy in these troublesome affections; and certainly it is more efficacious than the drugs generally employed.

It may be remarked that tar is useful in the same cases for which the spray of ipecacuanha wine is serviceable. The spray, we find, acts much more quickly, and, unlike tar, it lessens dyspnœa even before it improves cough or diminishes expectorations.

We have this year continued to carry on our observations with ipecacuanha wine spray, and with results confirmatory of the statements made in August last. We find, however, that some patients are very intolerant of ipecacuanha spray, which causes in them a good deal of irritation, and even tightness of breathing. It is advisable, therefore, at first to dilute the wine with one or two parts of water; a precaution especially needful for patients affected with much dyspnœa, with lividiy; for the spray may for some hours much intensify the difficulty of breathing and lividity, so as to alarm the patient and friends.

It may be not much out of place to mention here that, in several cases, we have found the spray very serviceable in non-febrile inflammatory sore throats, the mucous membrane being swollen and very red. We have found it useful, too, in hoarseness from congestion of the vocal cords. Where the hoarseness has lasted a few days only, or one or two weeks, the spray often speedily cures; but, where the hoarsness has presisted three months or longer, the spray even improves the voice considerably, but some hoarseness remains.—*British Med. Jour.*

